# How He Took His Bath

**Published:** 1874-12

**Authors:** 


How he took his Bath.—The Cape May *Wave* relates the following incident: "Among the excursionists that came from Wilmington by steamer was an unevenly-weighed man and wife—she, 300 avoirdupois; he, scarce 100 pounds would poise. Well, they must bathe, of course; what were the trips to Cape May without an ocean dip? A bath-house was secured. By tight squeezing our fat lady got into a bathing robe that was ready to burst at every step. Little husband girded about his body a woolen garment that fit like a shirt on a kildeer. Down they go, bold as a couple of whales, to the water; but just at the ocean's edge Puny suddenly halted, looked with awe on the furious billows, and then into the face of his determined three hundred. On her countenance were the words 'Come on!' On his trembling lips shivered the sounds, 'Oh, no!' The small specimen of diminutive husbandry feared to risk his diminutive portion of flesh and bones in the dashing foam, lest some unlucky billow might swallow him down like a snipe. 'You shall go in,' said the fat woman. 'I won't,' said Skinny, at the same time making frantic efforts to tear away; down he goes into the sand, scratching worse than a Kilkenny cat. Down drops the 300 upon terrified Bones, slick as a hawk upon a spring chicken. The sand flew, legs kicked, man screamed; yet in spite of all, the mammoth wife gathered her 100 pounds of furious sweetness in her arms, walked complacently into the biggest breakers, and kersouse she landed him headforemost in the sea; and as he popped up to the surface, half strangled, she pressed him to her bosom, saying, 'Now, honey, that's what you came all the way from Wilmington to enjoy.'"

